# Assessment of the Olfactory Function in Italian Patients with Type 3 von Willebrand Disease Caused by a Homozygous 253 Kb Deletion Involving *VWF* and *TMEM16B/ANO2*

**DOI:** 10.1371/journal.pone.0116483

**Published:** 2015-01-30

**Authors:** Valentina Cenedese, Massimo Mezzavilla, Anna Morgan, Renato Marino, Cosimo Pietro Ettorre, Maurizio Margaglione, Paolo Gasparini, Anna Menini

**Affiliations:** 1 Neurobiology Group, SISSA, International School for Advanced Studies, Trieste, Italy; 2 Institute for Maternal and Child Health, Istituto Di Ricovero e Cura a Carattere Scientifico “Burlo Garofolo” and Department of Medical, Surgical and Health Sciences, University of Trieste, Trieste, Italy; 3 Centro Emofilia e Trombosi, Azienda Ospedaliero-Universitaria Ospedale Policlinico Consorziale “Giovanni XXIII”, Bari, Italy; 4 Genetica Medica, Dipartimento di Medicina Clinica e Sperimentale, University of Foggia, Foggia, Italy; Duke University Medical Center, UNITED STATES

## Abstract

Type 3 Von Willebrand disease is an autosomal recessive disease caused by the virtual absence of the von Willebrand factor (VWF). A rare 253 kb gene deletion on chromosome 12, identified only in Italian and German families, involves both the *VWF* gene and the N-terminus of the neighbouring *TMEM16B/ANO2* gene, a member of the family named transmembrane 16 (*TMEM16*) or anoctamin (*ANO*). TMEM16B is a calcium-activated chloride channel expressed in the olfactory epithelium. As a patient homozygous for the 253 kb deletion has been reported to have an olfactory impairment possibly related to the partial deletion of *TMEM16B*, we assessed the olfactory function in other patients using the University of Pennsylvania Smell Identification Test (UPSIT). The average UPSIT score of 4 homozygous patients was significantly lower than that of 5 healthy subjects with similar sex, age and education. However, 4 other members of the same family, 3 heterozygous for the deletion and 1 wild type, had a slightly reduced olfactory function indicating that socio-cultural or other factors were likely to be responsible for the observed difference. These results show that the ability to identify odorants of the homozygous patients for the deletion was not significantly different from that of the other members of the family, showing that the 253 kb deletion does not affect the olfactory performance. As other genes may compensate for the lack of *TMEM16B*, we identified some predicted functional partners from *in silico* studies of the protein-protein network of TMEM16B. Calculation of diversity for the corresponding genes for individuals of the 1000 Genomes Project showed that TMEM16B has the highest level of diversity among all genes of the network, indicating that TMEM16B may not be under purifying selection and suggesting that other genes in the network could compensate for its function for olfactory ability.

## Introduction

The von Willebrand disease (VWD) is a hereditary coagulation abnormality in humans caused by qualitative (type 2 VWD) or quantitative defects (type 1 and type 3 VWD) of the von Willebrand factor (VWF), a protein involved in hemostasis [1]. The *VWF* gene is a large gene located on chromosome 12 (12p13.2) and has 52 exons spanning about 178 kb [2]. Type 3 VWD is caused by *VWF* mutations producing absence of VWF [3]. Inheritance of type 3 VWD is autosomal recessive and this disorder has a prevalence of about 0.5–3 individuals per million [4, 5]. Mutations of *VWF* causing type 3 VWD include nonsense mutations, splicing defects, insertions, and deletions (http://www.vwf.group.shef.ac.uk). Complete *VWF* deletions have first been found in Italian patients [6–8] and subsequently also in German patients [9, 10]. Schneppenheim et al [10] investigated patients with large deletions from unrelated German and Italian families. They found that a 253 kb deletion in chromosome 12 results in the deletion of the *VWF* gene and the deletion of the N-terminus of the neighbouring *TMEM16B* gene.

TMEM16B (also known as C12orf3, DKFZp434P102, anoctamin2 or ANO2) is one of the ten members of the protein family named transmembrane 16 (TMEM16). At least two members of this family, TMEM16A/ANO1 and TMEM16B/ANO2 have been shown to function as calcium-activated chloride channels [11–19]. TMEM16B/ANO2 is expressed in several tissues and organs, including retina, olfactory epithelium, pancreas, and salivary glands. In the olfactory epithelium, TMEM16B/ANO2 is expressed in the cilia of mature olfactory sensory neurons, where olfactory transduction occurs [15, 20–26]. However, the role played by TMEM16B/ANO2 in olfaction is controversial. Indeed, knockout mice for TMEM16B did not show any difference in olfactory sensitivity compared with wild-type mice [24] suggesting that this protein may be dispensable for normal olfaction, although another study [27] indicated that additional members of the CLCA family may also participate in the process of olfactory transduction together with TMEM16B, and substitute TMEM16B when it is absent.

Interestingly, Stephan et al. [15] refers to a possible impairment of the sense of smell in a German patient homozygous for the 253 kb deletion in chromosome 12, including *VWF* and the N-terminus of *TMEM16B*, suggesting an important role for TMEM16B in olfactory function. This observation, together with the evidence of expression of TMEM16B in the olfactory epithelium, prompted us to investigate the possible role of TMEM16B in the human sense of smell.

In this study, we identified a large Italian family that includes 4 patients homozygous for the 253 kb deletion (type 3 VWD patients) and measured the olfactory function of several members of the family, as well as of Italian healthy control subjects with similar sex, age and education. We found that the olfactory ability of the patients was significantly lower than that of the controls. However, also other members of the family, 3 heterozygous (type 1 VWD) for the deletion and 1 wild type, had a slightly reduced olfactory function indicating that cultural, social or other causative factors were likely to be responsible for the observed difference. If TMEM16B/ANO2 is involved in the sense of smell and is deleted in patients homozygous for the 253 kb deletion, we hypothesized that other genes may compensate for its lack. We therefore *in silico* studied the protein-protein network of TMEM16B/ANO2 and identified some predicted functional partners. These genes have been used to calculate the gene diversity in individuals belonging to the 1000 Genomes Project. Results indicate that TMEM16B/ANO2 has the highest level of diversity among all genes present in the network indicating that it may not be under purifying selection and thus, further suggesting that other genes may compensate for its function.

## Materials and Methods

### Subjects

This study was performed on 4 Italian siblings affected by VWD type 3 homozygous for the 253 kb deletion in chromosome 12 [10] and 4 members of the same family, 3 type 1 VWD (all heterozygous) and 1 unaffected (wild type homozygous). The mother was 67 years old and the 7 tested siblings (2 females and 5 males) were aged between 30 and 41 years. Their school education ranged between 5 and 8 years. A control group was constituted of 54 healthy Italian subjects, 29 females and 25 males, of age comprised between 21 and 57 years, and of a subgroup of 5 people with sex, age and education similar to those of the homozygous patients.

Genomic DNA was extracted from buccal swabs of the members of the family. In order to test the presence of the 253Kb deletion we performed deletion specific PCR as described by Schneppenheim et al [10]. Briefly, two sets of primers were used, one allowing the amplification of a product of 228 bp including the breakpoints of the deletion (Primer forward 5’-AAGAACCGAAGTCCCAGGAGAAAGGAAAG-3’, Primer reverse 5’-AGATTTCAGAGGCGTTCTAAAACTCACTC-3’) and the second one for the amplification of the wild type (279 bp) (Primer Forward 5’- GGAAAGTGGGATGGCGACAGAGCCTGAG- 3’, Primer reverse 5’-AGATTTCAGAGGCGTTCTAAAACTCACTC-3’). A standard PCR was carried out at 61°C, for 35 cycles using KAPA2G Fast ReadyMix PCR Kit (Kapa Biosystems), according to the manufacturer’s protocol. Healthy subjects carry only the wild-type sequence of 279 bp, homozygous patients show only the PCR product of 228 bp, while carriers present both fragments. All PCR products were then sequenced with 3500dx Genetic Analyzer (Life Technologies) to validate the nature of the deletion.

### Olfactory Testing

The Italian version of the University of Pennsylvania smell identification test [28] UPSIT, Sensonics, Haddon Heights, NJ, USA) was used to assess the ability to identify odorants. The Italian UPSIT test is a scratch-and-sniff test based on the forced-choice among four alternative odorants. Each subject was asked to scratch the microincapsulated odorants present in each page of four booklets with the tip of a pencil, provided in the kit. The four possible responses for each odorant were read to the subject, who indicated the name of the odorant more similar to the perceived smell. The total number of microencapsulated odorants was 40. The number of correct answers was calculated according to the test manual. A reduced and adapted version of the Italian UPSIT was used to evaluate the sense of smell in our subjects, as explained in the Results. Indeed, as previously pointed out, the names of some odorants reported in the UPSIT test did not match the common perception of those odorants by the Italian population [29, 30]. The same conclusions were obtained both by using the complete or the reduced and adapted version of the Italian UPSIT test.

### Ethical statement

Family members and healthy controls were recruited through the Medical Genetics Service of Children Hospital IRCCS-Burlo Garofolo, Trieste, Italy. All participants were informed about genetic tests and signed the appropriate Institutional consent form prepared according to our national rules and laws, in particular: i) art.10 and 22 of the Italian law 196/03 on privacy and following updates published on the Gazzetta Ufficiale n.65 of 19 March 2007, ii) National authorization n.2–2004 to conduct research activities on genetics, iii) Government authorization to referral research centers (an IRCCS in our National Health Care System) to perform genetic tests 02–2007. Finally, the study was approved by the Institutional Review Board (Comitato Tecnico Scientifico) of the Institute of Child Health IRCCS Burlo-Garofolo and conformed to the tenets of the Declaration of Helsinki.

### Protein-protein network and gene diversity

A protein-protein network indicating the predicted functional partners for TMEM16B/ANO2 was constructed using STRING v9.01 [31], a database of known and predicted protein interactions (http://string-db.org/).

Gene diversity for the genes involved in the above mentioned network was calculated using all the 1093 individuals from 1000 Genomes Phase 1 [32] taking into account all the single nucleotide polymorphisms (SNPs) or only the missense mutations using the method described in [33]. For each gene, gene diversity $1-\sum_{i=1}^{n}x_{i}^{2}$ [34], where *n* is the number of alleles, x_i_ the frequency of the *i*th allele.

### Statistical analysis

Data are presented as number of correct answers, and mean ± SEM. Statistical analyses were performed using Wilcoxon-Mann-Whitney test with R software.

## Results

### Olfactory testing

**Figure 1:**
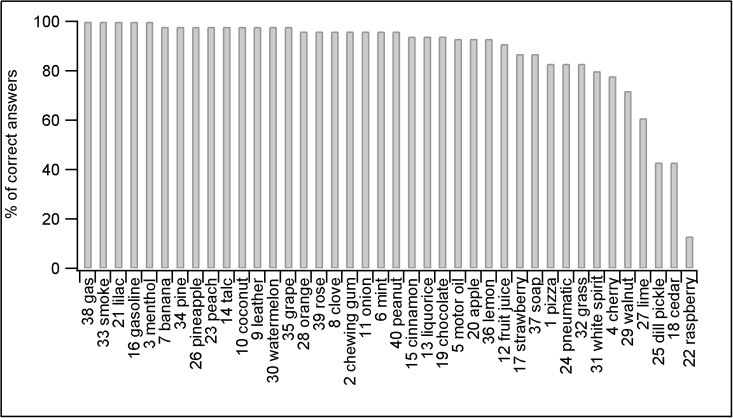
Distribution of answers to the Italian UPSIT test. Percentage of correct answers to the 40 odorants of the Italian version of the UPSIT smell identification test by the healthy subjects (n = 54).

As previous studies have reported that some of the odorants contained in the UPSIT booklets are not recognized by the Italian population [29, 30], we first tested the olfactory function of 54 healthy Italian subjects, 29 females and 25 males, of age comprised between 21 and 57 years, using two different test batches, with different expiration dates, of the Italian version of the UPSIT smell identification test. Among the 40 odorants present in the test, we found that 6 of them were not recognized by >20% of the subjects (Fig. 1). As the UPSIT test consists in a forced-choice among four possible responses, the probability to correctly identify the odorant by chance is 0.25. As in a previous study [29], we therefore discarded the 6 odorants that were not identified by more than 20% of the subjects, as the released substances did not correspond to the perception that our Italian test subjects had of the odorants indicated as the correct answer. In addition, one odorant indicated as “cuoio” (leather) was identified as “mela” (apple) by 92% of the test subjects. We therefore used “mela” (apple) as the correct answer for this odorant. We calculated the number of correct answers of the subjects by using our adapted Italian UPSIT test consisting of 34 odorants. Furthermore, we adapted the classification for olfactory function, based on 40 odorants, reported by Doty [28] by reducing the scale from 40 to 34, as follows: total anosmia 0–15, severe microsmia 16–19, moderate microsmia 20–23, mild microsmia 24–28, normosmia 29–34. On average, the number of correct answers of our healthy control subjects was 32 ± 0.2 (n = 54). 98% (53/54) of our test subjects were normosmic and 2% (1/54) were mild microsmic.

**Figure 2:**
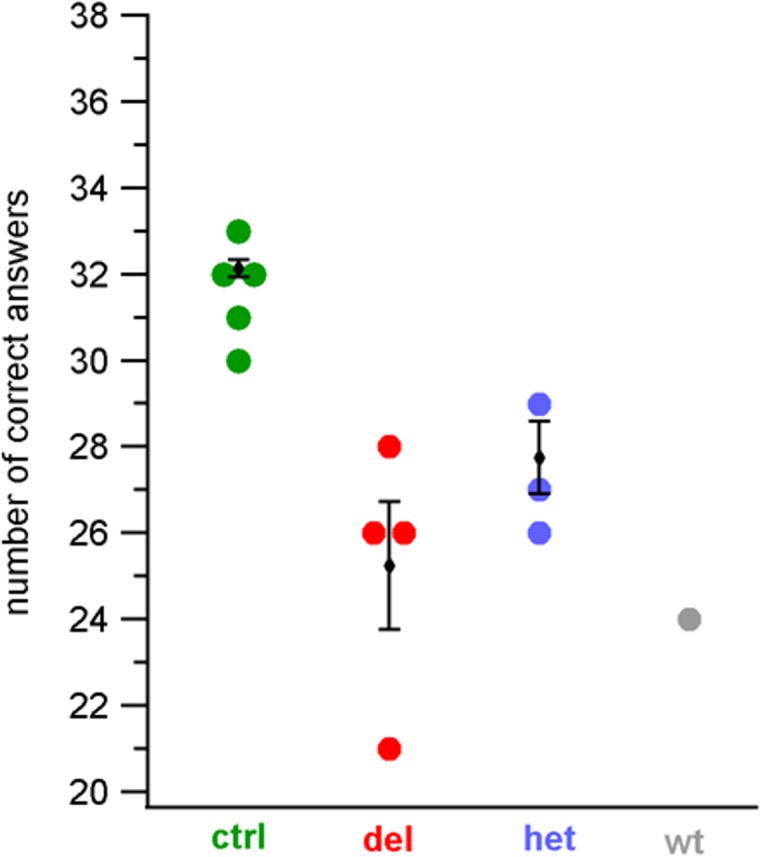
Assessment of the olfactory function with the adapted Italian UPSIT test. Number of correct answers to the 34 odorants selected for our adapted Italian UPSIT test for the subgroup of healthy subjects (indicated as control, “ctrl”, green, n = 5), or for members of the family homozygous for the 253 kb deletion in chromosome 12 (indicated as “del”, red, n = 4), heterozygous for the 253 kb deletion (indicated as “het”, blue, n = 3), wild type (indicated as “wt”, grey, n = 1).

We used the same test and analysis to assess the sense of smell of 4 siblings (1 female, 3 males) homozygous for the 253 kb deletion in chromosome 12 [10], and of a subgroup of control subjects (2 females, 3 males) with sex, age and education similar to those of the patients. As shown in Fig. 2, the UPSIT scores for the patients were 21, corresponding to moderate microsmia, and 26 or 28, corresponding to mild microsmia, whereas the scores of the control subjects ranged between 30 and 33, indicative of normosmia. On average, the number of correct answers of the 4 siblings was 25.3 ± 1.5 significantly lower than the value of 31.6 ± 0.5 (n = 5), obtained with the matched subgroup of control healthy subjects (p = 0.009).

Although this comparison indicates a significant decrease of the olfactory function in the patients homozygous for the deletion compared to the healthy subjects, we also tested four healthy family members to investigate whether the decrease in the sense of smell could have socio-cultural or other type of origins within the family. Three additional siblings plus the mother were tested being 3 heterozygous carriers of the deletion (1 male, 1 female and the mother) and one (male) wild type homozygous. The UPSIT scores of the 3 heterozygous subjects were 26, 27 and 29 (the mother), with the first two scores corresponding to mild microsmia, and the third one to normosmia. Moreover, the UPSIT score of the wild type homozygous brother was 24, again in the range of mild microsmia. On average, the number of correct answers of the 3 heterozygous subjects was 27.3 ± 0.9, not significantly different from the value of 25.3 ± 1.5 (n = 4), obtained with the family members who are homozygous for the deletion (p = 0.18).

Thus, these results show that the ability to identify odorants of the homozygous patients for the deletion was not significantly different from that of the other healthy members of the family, showing that the 253 kb deletion does not affect the olfactory performance.

It must be noted that these conclusions are not specific to the selection of 34 out of 40 odorants, as we obtained similar results also by taking into account all the 40 odorants contained in the Italian UPSIT booklets.

### Protein-protein network for TMEM16B/ANO2

**Figure 3:**
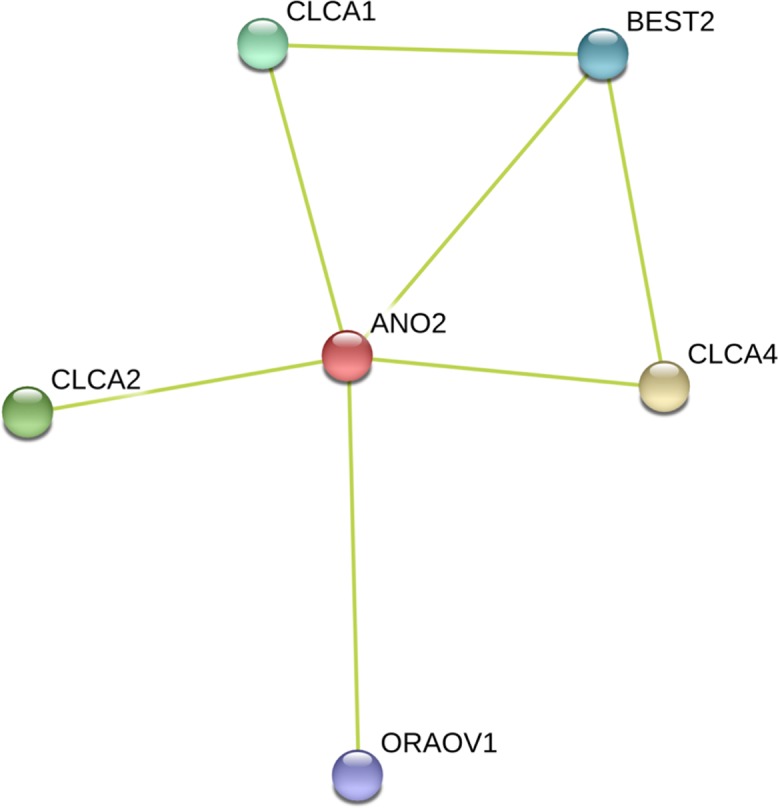
Protein-protein interaction network for TMEM16B/ANO2. Predicted functional partners obtained with the database STRING v9.01 (http://string-db.org/).

When a gene is deleted, it is possible that other genes compensate for its absence. It is therefore possible that a lack of TMEM16B/ANO2 is compensated by other interacting proteins. To identify the predicted functional partners, we constructed a protein-protein network using the database STRING v9.01 [31]. Fig. 3 shows that TMEM16B/ANO2 is predicted to interact with at least 5 partners, although it must be noted that these interaction partners were predicted only by textmining, as experiments are not available at present. CLCA1, CLCA2 and CLCA4 are members of the chloride channel accessory family named CLCA and may be involved in mediating calcium-activated chloride conductance. Both CLCA2 [25] and CLCA4 are expressed in olfactory sensory neurons [27] while mRNA for CLCA1 has been found in the olfactory epithelium of mouse embryo by *in situ hybridization* [35], but at present its localization to olfactory sensory neurons in unknown. BEST2, bestrophin 2, is known to form calcium-sensitive chloride channels and is expressed in the cilia of olfactory sensory neurons [14, 36, 37]. ORAOV1, oral cancer overexpressed protein 1, is expressed in olfactory sensory neurons (Supplementary Table S5 in [38]. Therefore CLCA2, CLCA4, BEST2 and ORAOV1 are all expressed in olfactory sensory neurons and may compensate for the lack of TMEM16B/ANO2.

### Gene diversity in the TMEM16B/ANO2 network

One aspect that highlights the deleterious consequences of a specific allele is the evidence of purifying selection, which acts against mutations that have deleterious effect. The effect of this selection consists in reduced gene diversity in the selected locus, in particular affecting the missense sites (which cause a change in amino acid residue), for this reason we estimated gene diversity for missense SNPs and SNPs at which there were no amino acid change (5’ and 3’ UTR, synonymous sites, intron variants).

**Figure 4:**
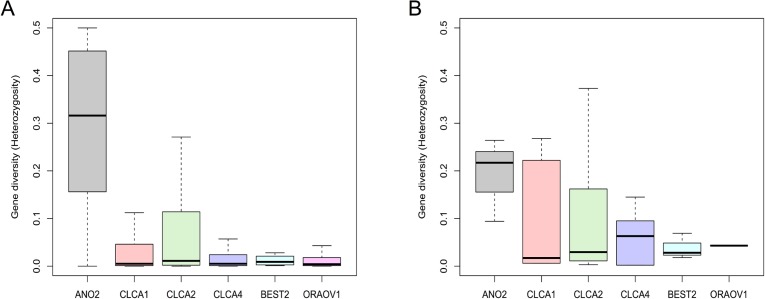
Gene diversity for *TMEM16B/ANO2*. Gene diversity calculated in individuals of the 1000 Genomes Project using all the SNPs in the genes (**A**) or only the missense mutations (**B**). *TMEM16B/ANO2* showed the highest gene diversity among the indicated genes both in A (p = 2.2e-16) and in B (p = 0.042).

To determine gene diversity for the genes involved in the network of Fig. 3, we used data from all the 1093 individuals from the 1000 Genomes Project Phase 1 [32]. We investigated gene diversity using all SNPs and found that *TMEM16B/ANO2* has the highest diversity among the genes in the network (Fig. 4A). In addition, we also calculated gene diversity taking into account only the missense mutations and confirmed that *TMEM16B/ANO2* has the highest level of gene diversity, further suggesting the absence of constrains in variation in SNPs causing amino acid changes (Fig. 4B).

These results indicate that *TMEM16B/ANO2* may not be under purifying selection whereas CLCA1, CLCA2, CLCA4, BEST2 and ORAOV1, that presented a much lower gene diversity, may be under purifying selection and thus should play a more relevant role that cannot be compensated by other genes.

## Discussion

In this study, we measured the odorant identification ability of Italian patients affected by type 3 VWD caused by a 253 kb deletion in chromosome 12 producing the deletion of the *VWF* gene and of the N-terminus of the neighboring *TMEM16B* gene. We conducted this study because the only observation about the sense of smell in a patient homozygous for the 253 kb deletion was the personal communication by Drs. Roswitha Eisert and Reinhard Schneppenheim, reported by [15], describing the following observations from an interview with the widow of a patient: “1) her husband never complained about the meals even if they were burnt. 2) When [the patient] was cooking he was possibly not noticing when the potatoes were burnt. 3) [His] use of perfume was sometimes so extreme that his wife wondered how he could stand it. 4) He never mentioned the smell of flowers or ‘rural’ smells.”

We first compared the olfactory function of a healthy subgroup of Italian subjects with that of the group of patients, with similar age, sex and education. Our results reveal that homozygous patients for the deletion have a slightly reduced ability in identifying odorants compared to healthy subjects. The UPSIT scores for 34 odorants of 5 healthy subjects varied from 30 to 33, whereas those for the 4 homozygous patients were 21, corresponding to moderate microsmia, 26 (for 2 subjects) and 28, indicating mild microsmia. However, when we extended the assessment of the olfactory function to other members of the same family, including 3 heterozygous subjects for the deletion, carrying a type 1 VWD, and 1 wild type, we found that the UPSIT score of the wild type subject was 24 (mild microsmia) and the scores of the 3 heterozygous subjects were 26, 27 and 29 (the mother), with the scores of the 2 siblings in the range of mild microsmia and the score of the mother in the range of normosmia. Although one homozygous patient had a rather low score of 21 (moderate microsmia), the average value for the 4 patients was not significantly different from that of the other members of the family, indicating that the lower UPSIT scores compared to those of the subgroup of healthy subjects is likely due to socio-cultural or familiar origins, which we have not further investigated, but that are unrelated to the 253 kb deletion. A larger number of patients would allow a better estimation of the olfactory function, but it must be noted that the type 3 VWD is a rare disease, with a prevalence of about 0.5–3 individuals per million [4, 5], and that the 253 kb deletion has only been found in some Italian and German families. Therefore, it has been an extraordinary privilege to have the consent of several members of the large Italian family containing subjects with the 253 kb deletion to have their olfactory function assessed with the UPSIT test and to have buccal swabs taken for the subsequent search of the deletion.

The similar olfactory abilities among members of the Italian family comprising patients homozygous for the 253 kb deletion, together with the observation that the lowest UPSIT score for homozygous patients was in range in moderate microsmia and not in the range of severe microsmia or of anosmia, may indicate that TMEM16B is not necessary for normal olfaction, in agreement with a previous study on knockout mice for TMEM16B [24]. However, it is important to note that, as previously discussed by [10], the phenotype related to the *VWF* deletion is well defined by clinical symptoms and laboratory parameters and corresponds to type 3 VWD, whereas at present a phenotype associated to the *TMEM16B* deletion has not been identified. In addition, since the 253 kb deletion involves only the N-terminus of *TMEM16B* gene, we cannot exclude the possibility that alternative splicing could produce a functional protein isoform. Indeed, previous studies have shown that another member of the TMEM16 family, *TMEM16A*, has transcripts missing the first N-terminal region of 116 amino acids [11, 19, 39, 40]. Splice variants for TMEM16B have also been identified, including the presence of an alternative starting exon that resulted in a shortened N-terminus [15, 41]. Thus, at present we cannot exclude the possibility that patients homozygous for the 253 kb deletion express a TMEM16B isoform that may have some different functional properties.

If TMEM16B is not functional, there is the possibility that other genes could compensate for the absence of TMEM16B. By *in silico* investigation of the protein-protein network for TMEM16B [31], we identified 5 predicted functional partners, 4 of which have been shown to be expressed in olfactory sensory neurons: CLCA2, CLCA4, BEST2 and ORAOV1 [25, 27, 35–37]. A previous study in rat olfactory epithelia reported molecular biological and immunochemical evidence that CLCA2 and CLCA4 are expressed in the cilia of a subset of olfactory sensory neurons [27]. These authors suggested the possibility that these calcium-activated chloride conductances may participate to olfactory transduction together with TMEM16B, and could substitute TMEM16B when it is absent. BEST2 is another calcium-activated chloride channel expressed in the cilia of olfactory sensory neurons [36]. ORAOV1 is known to be overexpressed in oral cancer and it is also expressed in olfactory sensory neurons [38], although its function is at present unknown.

We determined gene diversity for the genes involved in the network using individuals from the 1000 Genomes Project and found that TMEM16B has the highest gene diversity among the other genes, both when diversity was calculated using all SNPs and only taking into account the missense mutations. These results may indicate that TMEM16B is not under purifying selection, highlighting the evidence that there are no constraints in this gene and providing an indirect clue about the presence of other genes, such as BEST2, CLCA1, CLCA2, CLCA4, or ORAOV1 that could compensate for its function for olfactory ability.
